# Multifaceted Therapeutic Potential of Plant-Derived Exosomes: Immunomodulation, Anticancer, Anti-Aging, Anti-Melanogenesis, Detoxification, and Drug Delivery

**DOI:** 10.3390/biom15030394

**Published:** 2025-03-10

**Authors:** Arzu Zeynep Karabay, Jaleh Barar, Yalda Hekmatshoar, Yalda Rahbar Saadat

**Affiliations:** 1 Department of Biochemistry, Faculty of Pharmacy, Ankara University, 06560 Ankara, Türkiye; akarabay@ankara.edu.tr; 2 Department of Pharmaceutical Sciences, College of Pharmacy, Nova Southeastern University, Fort Lauderdale, FL 33328, USA; jbarar@nova.edu; 3 Department of Medical Biology, Faculty of Medicine, Altinbas University, 34217 Istanbul, Türkiye; yalda.hekmatshoar@altinbas.edu.tr; 4 Kidney Research Center, Tabriz University of Medical Sciences, Tabriz 5165665811, Iran

**Keywords:** plant-derived extracellular vesicle, exosome, immunomodulation, anticancer, anti-aging, anti-melanogenesis, detoxification, drug delivery, therapy

## Abstract

Most eukaryotic and prokaryotic cells have the potential to secrete a group of structures/membrane-bound organelles, collectively referred to as extracellular vesicles (EVs), which offer several advantages to producer/receiver cells. This review provides an overview of EVs from plant sources with emphasis on their health-promoting potential and possible use as therapeutic agents. This review highlights the essential biological effects of plant-derived extracellular vesicles, including immune modulation, anticancer activities, protection against chemical toxicity and pathogens, as well as anti-aging, anti-melanogenesis, and anti-arthritic effects, along with ongoing clinical studies. Evidence revealed that plant-derived EVs’ contents exert their beneficial properties through regulating important signaling pathways by transferring miRNAs and other components. Taken all together, the data proposed that plant-derived EVs can be utilized as nutritional compounds and therapeutic agents, such as drug carriers. However, this emerging research area requires further in vitro/in vivo studies and clinical trials to determine the exact underlying mechanisms of EVs’ positive health effects in treating various diseases.

## 1. Introduction

**Figure 1:**
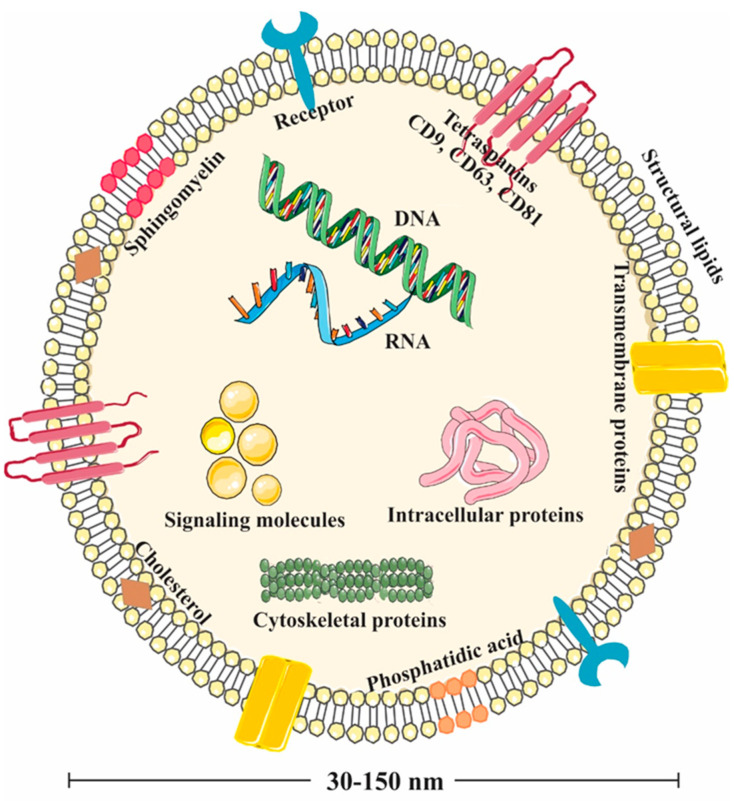
Schematic representation of an exosome’s structure. Exosomes are nanovesicles lined by a lipid bilayer, which encompass proteins, lipids, nucleic acids, etc., and express the surface ligands and receptors. DNA, deoxyribonucleic acid; RNA, ribonucleic acid.

Almost all cell types have the potential to secrete extracellular vesicles (EVs) enclosed by a lipid bilayer to transport their intracellular content (e.g., cytosolic proteins, lipids, and genetic materials) to interconnect with each other and even interspecies communications. These EVs include exosomes and microvesicles [1,2]. Exosomes (Figure 1) are lipid-bilayer membrane nanovesicles with a diameter ranging from 30 to 150 nanometers that derive from multivesicular bodies (MVBs) and plasma membrane fusion [3,4,5]. A wide variety of cells (e.g., immune (dendritic and mast cells), epithelial, and cancer cells) are capable of exosomal release under both physiological and pathological conditions, and the emitted exosomes have been found in different biological fluids (blood, saliva, urine, semen, and breast milk) [6,7,8]. These structures, which are considered to be extracellular messengers, have recently been identified in different food sources. The molecular organization of the exosomes preserves them in harsh environments (such as hydrolytic enzymes, etc.) exposed in biological fluids and surroundings, which in turn results in the safe delivery of their cargo into recipient cells [9,10]. These so-called extracellular messengers exert an important role in cell–cell communication through alterations in gene regulation and protein expression in target cells [11,12]. It has been shown that exosomes are capable of passing the blood–brain barrier (BBB). Additionally, the long circulation time and high bioavailability make them intriguing alternatives for therapeutic applications. Several lines of evidence suggested that PDEVs’ uptake is greatly dependent on the cell type. For instance, grape-derived EVs’ uptake highly relied on both micropinocytosis and clathrin-dependent pathways for entry into macrophages. On the other hand, ginger EVs’ entry to the hepatocytes depends on micropinocytosis; however, its uptake into Colon-26 and HT-29 cells was through phagocytosis. Additionally, the state of the recipient cells may affect the PDEVs’ uptake [3]. In this regard, Zhuang et al. explained that entry of the ginger-derived EVs into the liver was higher in mice fed with an ethanol diet in comparison to the mice fed with a normal diet [13]. In addition to the effects of recipient cell types on exosome uptake, the extraction method and the particle size distribution after extraction may also influence exosome uptake [14].

Besides the beneficial properties of EVs, there are some challenges regarding their utilization. For instance, a single cell can produce various types of vesicle subpopulations, thus dissociation among the cocktail of subpopulations remains a challenge in the field of EVs [15]. Different terms (for instance, nanoparticles, exosome-like nanoparticles, nanovesicles, and nanoshuttles) have been used to define these structures; however, in the present review, we extensively use both “extracellular vesicles” and “exosomes” to incorporate all the terms mentioned before. Here, we aim to summarize studies regarding plant-derived EVs, their functions, and future applications in health and disease.

## 2. Biogenesis of Exosomes

**Figure 2:**
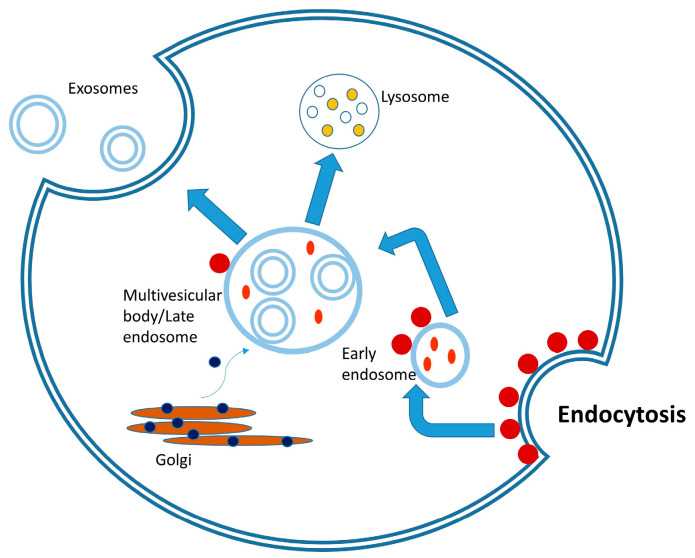
Biogenesis of exosomes. Internalized and trans-Golgi network-derived cargos are sorted into early endosomes, which mature into multivesicular bodies (MVBs). MVBs fuse with the plasma membrane, releasing exosomes.

The first step in the biogenesis of the exosome structure is the bud formed by the invagination of the plasma membrane toward the cytosol (Figure 2) by taking a portion from the extracellular fluid. This event leads to the formation of an early endosome structure [16]. It has been observed that the plasma membrane of the early endosome carries various membrane-bound proteins and the proteins in the lumen of the cytosol and extracellular fluid the early endosome originates from. In the process following the formation of the early endosome, inward invaginations of the endosomal membrane, mediated by various protein complexes, lead to the formation of intraluminal vesicles in the early endosome structure and, subsequently, the structure called the late endosome or multivesicular body is formed with the maturation of the endosome [17].

When multivesicular vesicles fuse with the plasma membrane and release their cargo into the extracellular space, the intraluminal vesicles are called exosomes [18]. Tetraspanins in the exosome membrane play a role in selecting and loading the cargo containing the signaling proteins, whereas membrane glycoproteins play a role in attachment to the recipient cell. On the other hand, lipid raft components, such as cholesterol, sphingomyelin, and phospholipids, play a role in regulating fluidity in certain regions [19,20,21].

The components carried by the exosome include growth factors, cytokines, HSPs, cytoskeletal proteins involved in budding, nucleic acids, and the ESCRT system (Alix, TSG101), which plays a role in identifying the proteins in the exosome cargo and loading them into exosomes. The ESCRT system, which consists of members such as ESCRT 0-1-2-3 and adaptor proteins Alix and syntenin, ensures that the cargo undergoes the correct conformational changes and passes into the exosome lumen. The ESCRT system identifies the proteins to be selected or left behind through protein labeling. Accordingly, ubiquitin-labeled proteins bind to the ESCRT complex, and Alix is activated to transport these proteins into the lumen, like a shuttle system. This pathway is one of the active mechanisms in the loading of exosomes with cargo [22]. Tetraspanins play a similar function in cargo specification and loading through a pathway independent of the ESCRT system [23]. Lipid rafts in early endosomes alter the membrane fluidity and recruit specific proteins to the membrane structure that will function in budding, which also occurs as an ESCRT-independent mechanism [24]. Once released and taken up by recipient cells, exosomes can exert downstream effects, such as changes in cell phenotype and behavior [25]. There are several pathways by which exosomes are taken into the cell, including endocytosis, fusion, and receptor signaling [26].

Nanovesicles of plant origin have also been found to stimulate the passage of the cell wall, transport proteins, lipids, nucleic acids, and metabolites. The content of these vesicles varies depending on the cell from which they are released [27]. Plant-derived nanovesicles are rich in transmembrane proteins, like tetraspanins, with roles such as movement, cargo selection, fusion, biogenesis, and recognition of nanovesicles. It has also been reported that they contain plant-specific secretory syntaxin, which is called PEN 1 and has various roles, including biogenesis of the plant wall and defense against pathogens [28,29]. The biogenesis of plant-derived nanovesicles is similar to exosome biogenesis. In addition to the biogenesis pathways of tetraspanins and PEN 1, exosomes are formed by a third pathway mediated by double-membrane structures, called EXPO (exocyst-positive organelles), similar to autophagosomes. In biogenesis, the formation of intraluminal vesicles within multivesicular bodies during late endocytosis occurs, which is followed by release of exosomes by fusion of these vesicles with the plasma membrane [30].

## 3. EVs Derived from Natural Green Nano-Factories

Plant-derived extracellular vesicles (PDEVs)—which originate from MVBs—were demonstrated in 1967 and play major roles in intercellular as well as interspecies interactions (through transferring plant material to various cells) [3,7,31]. PDEVs display exclusive features, such as safety, a significant possibility for large-scale production, targeted drug delivery, and therapeutic properties against different diseases [32]. Additionally, these vesicles have the capability of transporting mRNAs, miRNAs, bioactive lipids, and proteins to human and animal cells [7,33,34,35,36]. People who routinely consume a variety of edible plants (fruit and vegetables) may face several PDEVs with beneficial health effects [6]. Research has revealed differences in the content of mammalian and plant exosomes [37]. In line with this, it has been shown that mammalian exosomes comprise 100–300 miRNAs and over 1000 proteins, while EVs derived from grape (GELNs) are composed of 28 identified proteins and approximately 100 miRNAs [9].

### 3.1. Lipids in PDEVs

Regarding the lipid profile, structural analysis of mammalian exosomes revealed that they usually contain cholesterol and sphingomyelin, however, they have low amounts of phosphatidylethanolamine and phosphatidic acid (PA; mitogenic phospholipid) [9]. Contrary to mammalian exosomes, GELNs were enriched for phospholipids (up to 98%, ~50% of which was PA), and the remaining 2% were composed of typical plant lipids (e.g., galactolipids, such as digalactosyldiacyl-glycerol and monogalactosyldiacyl-glycerol). PA interacts with the mammalian target of rapamycin (mTOR), which in turn activates cell growth and proliferation. Besides, PA is extremely fusogenic when calcium is present, thus inducing inter-vesicular fusion [9,38,39,40]. PA induces cytoskeleton rearrangement and might be involved in vesicular trafficking/endocytosis [3]. Another vital role of PA was determined by studies on PA-depleted ginger EVs, which indicated PA’s role in maintaining the duration and amount of PDEVs’ accumulation in the gut and their utilization by various bacteria [41]. The lipidomic data illustrated differences between various PDEVs. The grapefruit-derived exosome-like nanoparticles (GDNs) were enriched for phosphatidylethanolamine and phosphatidylcholine. The diversity of the lipid profile may play a major role in interspecies communication throughout the mammalian gastrointestinal tract (GIT) [7,42]. In a recent study, the contents of ginger-, lemon-, grapefruit-, and grape-derived EVs were compared, and lipids belonging to six different categories were screened in exosomes, including glycerolipids, sphingolipids, sterol lipids, fatty acids, glycerophospholipids, and prenol. The results showed that exosomes of ginger, grape, and lemon origin contained more glycerolipids, while exosomes of grapefruit origin had higher glycerophospholipid content. It was determined that glycerophospholipid content was lower and the ratio of fatty acids was higher in EVs of ginger origin. Differences were also determined between the ratios of fatty acid contents in exosomes. Here, 465 lipid species were commonly detected in all 4 exosome types, whereas 29, 400, 87, and 40 lipid species were unique for exosomes of grapefruit, ginger, lemon, and grape origin, respectively. Even if PDEVs carry some common lipid species, each plant may have its unique lipid profile [43].

Lipidomic analysis of exosome-like vesicles obtained from edible tea flowers revealed that the most abundant lipids were phosphotidylcholine, triglycerides, and phosphotidylethanolamine, respectively [44]. Nanovesicles obtained from the gel and bark of the *Aloe* plant have been reported to contain ceramides, mainly glycosylceramide, phosphatidic acid, and phosphotidylcholine [45]. In another study, it was determined that orange-derived nanovesicles contained phosphotidylethanolamine in the highest concentration, followed by phosphotidylcholine and phosphotidic acid. Diacylglycerol and fatty acids, such as palmitic acid, oleic acid, linoleic acid, and linolenic acid, were also found in the vesicles [46]. The five most abundant lipids in exosome-like nanoparticles originating from turmeric plants were phosphotidylethanolamine, triglyceride, phosphotidylinositol, phosphotidylcholine, and digalactosylglycerol, respectively [47].

Differences between mammalian and plant-derived extracellular vesicles include the fact that M-EVs are rich in cholesterol and sphingomyelin, while PDEVs are cholesterol-free and contain phosphotidic acid, phosphotidylcholine, phosphotidylethanolamine, and digalactosyl monoacyl glycerol. These differences are thought to be important in the interspecies communication of PDEVs and in the transfer of vesicles to recipient cells [48].

### 3.2. Proteins in PDEVs

In the period following the discovery of exosomes, studies on their protein content have also gained momentum. With the development of proteomic analyses that allow the identification of proteins on a large scale, obtained data can be accessed from various databases. The Exocarta database (http://www.exocarta.org/, (accessed on 25 February 2025)), where data from mammalian exosomes are stored, has been merged with another database, called Vesiclepedia (http://www.microvesicles.org/), and comprehensive findings, including various types of extracellular vesicles, have been shared. Evpedia.info (https://evpedia.info/evpedia2_xe/), a database of non-mammalian extracellular vesicles of different sizes, is also among the databases that provide useful findings for vesicle researchers. The databases contain information on the proteins, lipids, mRNA, miRNA, and metabolites found in vesicles.

Proteins found in extracellular vesicles of plant origin are used in defense and stress responses of the plant, intracellular trafficking, and signaling events, such as transport and fusion. It has been determined that the proteins in the extracellular vesicles obtained from *Arabidopsis thaliana* are involved in biotic and abiotic stress responses of the plant. RPM1-interacting protein RIN4 and various RIN4-related proteins, as well as glucosinolate transporter PEN3, a member of defense-related proteins, were identified among the proteins involved in stress responses and immunity. The extracellular vesicle proteome was also identified to contain phospholipase Dα (PLDα), PLDδ, Annexin1, and glutathione S transferase PHI2, which are involved in reactive oxygen species signaling and oxidative stress responses, respectively. Proteome analysis showed that they also richly contain syntaxins involved in plasma membrane transport, such as PEN1 involved in membrane traffic and SYP71 involved in immunity. RAB GTPases involved in vesicle transport and fusion, such as PATELLIN1 (PATL1) and PATL2, and ATPases involved in the transport of water, ions, and other compounds are also among the proteins identified in vesicles [29].

In another study, which analyzed extracellular vesicles isolated from *Craterostigma plantagineum*, cell-wall-associated proteins, such as 1,3-β-glucosidases, pectin esterases, polygalacturonases, β-galactosidases, and β-xylosidase/α-L-arabinofuranosidase, aquaporins, such as plasma membrane intrinsic protein 1C and aquaporin PIP2-7-like protein, proteins involved in oxidative reactions, such as reticuline oxidase-like protein, HSP 70-like chaperones, and various proteins involved in stress responses were identified [49].

Protein profiles in vesicles originating from four citrus species revealed that ATPase 10, HSP90, and patellin were the commonly detected proteins. In addition, two gripping plant proteins, Patellin-3-like and clathrin heavy chain, which are known to mediate polarity, movement, and endocytosis, respectively, exhibited abundance in all four of the citrus vesicles. Chaperon proteins HSP70 and HSP80, and metabolic proteins 14-3-3, G3PD, and FBA6, were also highly represented in both micro- and nano-vesicles derived from citrus species. Aquaporin was another protein with high enrichment in the nanovesicle fraction of citrus plants and ginger [7,50], whereas hydrolases and antioxidant enzymes were also highly expressed in citrus vesicles [50]. Extracellular vesicles isolated from tomato roots were reported to contain the 14-3-3 protein family, actin, calmodulin, annexins, aquaporins, calreticulin, and fatty acid binding proteins, and were similar to the protein profile of vesicles of plant and animal origin. In addition, plant cell membrane-specific H+-ATPases, nitrate, and phosphate transporters were also enriched in vesicles [51].

### 3.3. Metabolites in PDEVs

Extracellular vesicles of plant origin contain different metabolites depending on the plant species and the part of plant from which the vesicles were isolated. These metabolites include alkaloids, flavonoids, phenolic substances, and various other active compounds [52]. Most of the metabolites have been reported to be secreted to enable the plant to protect itself against pathogens, as well as mediating biological effects, such as anti-inflammation, in other cells [42,52].

Examples of metabolites released from extracellular vesicles of plant origin include vitamin C from citrus plants [53] and strawberry [54], naringin from citrus fruits [55], gingerol from ginger [56], cannabidiol from cannabis [57], trans-δ-viniferin from grapes [58], anthraquinones from *Aloe* sp. [59], curcumin from turmeric [47], and phenolic acids, flavonoids, amino acids, terpenoids, and tannins from pomegranate [60]. Studies have also shown that these metabolites exhibit important biological effects in cells targeted by vesicles, including suppression of oxidative stress, inhibition of phototoxicity, anti-inflammatory, and anticancer effects.

### 3.4. miRNAs in PDEVs

PDEVs contain various types of nucleic acids, such as miRNAs, sRNAs, DNA, and other non-coding RNAs. Among these nucleic acids, miRNAs, which modulate cell signaling through gene expression changes in the target cell, have received the most attention [61]. Many pieces of research focus on elucidating the role of plant-derived miRNAs as messengers of intercellular communication [62,63,64]. Interestingly, there are probable mechanisms for plant miRNA trafficking pathways in the GIT. First, the presence of transmembrane proteins, which can form a channel to permit passive diffusion, is suggested. Another possible mechanism may involve the endocytosis of miRNAs from the lumen. The last mechanism by which plant miRNAs in food can transfer to the end organs of the mammals incorporates miRNAs packaged into the MVs [62].

#### Stability of Plant-Derived miRNAs

Plant-derived miRNAs may overcome various challenges while passing through food sources to reach the target organ of the animals. The extreme surroundings in the mammalian GIT (hydrolytic enzymes, phagocytosis, and a low pH) entail a stable structure of miRNAs to preserve them from degradation before entering the target cells [62]. Methylation of the 2′-hydroxyl group of the 3′-terminal nucleotide of the plant miRNAs prevents 3′-end uridylation and 3′-to-5′exonuclease digestion, which in turn results in a slower degradation rate of the plant miRNAs and, consequently, contributes to the stability of plant miRNAs in vivo [62,65]. It has been proposed that the stability of miRNAs may probably be related to the unique sequences of the molecules and their high guanine cytosine (GC) content [65,66]. Zhang and colleagues revealed that plants (such as rice, Chinese cabbage, wheat, and potato) could deliver the miR156a and miR166a, even after cooking; nevertheless, the quantity was low. Furthermore, exogenous plant miRNAs (for instance, miR168a, which is found in large quantities in rice) were present in the sera and tissues of different animals that utilized plants as food sources. In addition, they reported that miR168a could bind to the human/mouse low-density lipoprotein receptor adapter protein 1 (LDLRAP1) mRNA and, as a consequence, significantly reduced the LDLRAP1 expression in the liver, and decreased LDL removal from mouse plasma. The results of their study supported the hypothesis that exogenous plant miRNAs in food can modulate the expression levels of target genes in mammals and function in a cross-kingdom manner [67]. Furthermore, Zhou and colleagues performed a study on Chinese herb honeysuckle (*Lonicera japonica*) miRNA—miR2911—which directly targets influenza A viruses (IAVs). They showed that feeding mice with honeysuckle resulted in accumulation of miR2911 in peripheral blood and the lungs. Additionally, they stated that the miR2911 remains intact during the boiling process and could move throughout the GIT and accumulate in the lung. Data from their research illustrated that miR2911 suppresses the replication of IAVs (e.g., H1N1, H5N1, and H7N9) through binding the polymerase basic protein 2 (PB2) and nonstructural protein 1 (NS1) genes in the lung, and subsequently inhibiting death [68]. In another study, exosome-like nanovesicles were isolated from *Brucea javanica*, a medicinal plant known to be effective against various different cancer types, such as GI cancer and lung cancer, and were tested in 4T1 triple-negative breast cancer (TNBC) cells. It was determined that 10 functional miRNAs were transferred from the vesicles to 4T1 cells, and these miRNAs induced caspase-dependent apoptosis by suppressing the phosphatidylinositol 3 kinase (PI3K)/protein kinase b (Akt)/mTOR pathway, an important pathway in the survival of cancer cells. More importantly, it has been shown that BF-derived exosomes retain their stability and exhibit the same anticancer activity when stored at −80 °C for a year [69]. In another study, it was suggested that the ata-miR156c-3p carried by exosome-like nanovesicles isolated from the *Lycium ruthenicum* Murray plant protects PC12 cells against Aβ-induced toxicity and may reduce the expression of Alzheimer’s-related genes, such as integrin beta-3 (ITGB3) and platelet-derived growth factor receptor beta (PDGFRB), by binding to their 3′-UTR regions. Nanovesicles originating from the *L. ruthenicum* Murray plant maintain their stability during cryopreservation because they carry monogalactosyldiacylglycerol, which plays a role in the stability of phospholipid liposomes [70]. In another study, *Portulaca oleracea* L., which has both food value and medicinal properties, was tested. Histological evaluations and analysis of the colonic epithelial barrier, proinflammatory cytokine levels, and immune cell filtration revealed that exosome-like nanoparticles of *P. oleracea* L. origin exhibited protection in a dextran sulfate sodium (DSS)-induced C57BL/6 ulcerative colitis mouse model. In the model, IL-10-/- mice treated with nanoparticles surprisingly did not develop colitis. It was also observed that the nanoparticles increased beneficial bacterial species, such as *Lactobacillus reuteri*, in the microbiota of mice. It was also determined that nanoparticles originating from *P. oleracea* L. concentrated in the inflamed colon tissue instead of other organs and provided specific targeting, and did not cause any toxicity in vital organs and exhibited good stability in the GIT [71]. All these studies showed that plant-derived extracellular vesicles can transport the miRNAs they carry to target sites without disrupting their structure.

### 3.5. Therapeutic Approaches of PDEVs

Several lines of evidence demonstrated health-promoting effects of EVs isolated from diverse plant species [72,73]. Some of the studies conducted to date are discussed in this section.

#### 3.5.1. Immunomodulatory Properties

##### Intestinal Anti-Inflammatory Activity

**Figure 3:**
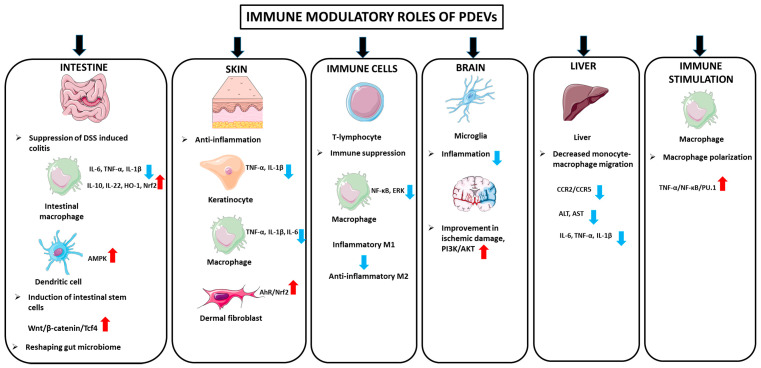
Immune modulatory roles of PDEVs. Plant-derived extracellular vesicles (PDEVs) contribute to immune modulation across various systems. PDEVs have been reported to inhibit inflammatory signals in the intestine, skin, immune cells, brain and liver. On the contrary, immune enhancer functions of PDEVs have also been reported.

PDEVs may contribute to the modulation of immune responses (Figure 3). Ju and colleagues isolated and characterized grape exosome-like nanoparticles (GELNs) from crushed grapes, which are composed of 96 miRNAs. GELNs passed through the intestinal mucus barrier and significantly led to the induction of leucine-rich repeat-containing G protein-coupled receptor 5n (Lgr5) intestinal stem cells via the Wnt/β-catenin pathway. For assessing the GELNs’ role in the induction of intestinal stem cells, they blocked the β-catenin-mediated signaling pathways of GELN recipient cells, and the results indicated that it may lead to a reduction in the formation of Lgr5+ stem cells. Furthermore, administration of GELNs orally resulted in induction of intestinal stem cells and subsequently protected the mice from DSS-induced colitis [4]. Using different mice models, Zhang et al. demonstrated that exosomes derived from ginger led to amelioration of acute colitis, enhanced intestinal repair, and inhibited chronic colitis and colitis-associated cancer (CAC). The mechanisms by which ginger-derived exosomes exert healing effects in colitis models were regarded as increased survival and proliferation of intestinal epithelial cells (IECs), decreases in the pro-inflammatory cytokine production (e.g., TNF-a, IL-6, and IL-1β), and enhanced production of the anti-inflammatory cytokines (IL-10 and IL-22) [7]. In another study, Deng et al. investigated the role of broccoli-derived nanovesicles (BDNs) in the regulation of intestinal immune homeostasis by targeting dendritic cells (DCs) in mice colitis models. BDNs exert anti-inflammatory properties by prevention of DC induction and activation of adenosine monophosphate-activated protein kinase (AMPK) in DCs [74]. Further, Wang et al. indicated that grapefruit-derived nanovesicles (GDNs) resulted in attenuated DSS-induced mouse colitis through selective uptake by intestinal macrophages. The GDNs exert these beneficial effects by upregulation of the heme oxygenase-1 (HO-1) and downregulation of IL-1β and TNF-α in intestinal macrophages [42]. In parallel with these findings, garlic-derived exosome-like nanovesicles suppressed IL-6, IL-1β, TNF-α, and IFN-γ levels, TLR4 and Myd88 pathways in LPS-treated Caco2 cells, and the dextran sulfate-treated mouse colitis model. When the mechanism of action was examined, it was determined that the exosome-like nanoparticles inhibited TLR4 by binding to the 3′ UTR of TLR4, especially through han-miR3630-5p [75]. Studies investigating the effects of EVs originating from different plants on colitis have continued until recently. In one of these studies, EVs originating from *Momordica charantia* have been reported to suppress oxidative stress and inflammation and exhibit protective effects in a C57BL/6 mouse model of ulcerative colitis [76]. It has been reported that *Houttuynia Chordata*, a traditional medicinal plant, decreased inflammatory cytokines, such as IL-6 and TNF-α, and increased the level of anti-inflammatory IL-10 and various tight junction proteins in a dextran sulfate sodium-induced colitis model [77]. Exosomes originating from tomato plant have been suggested to reshape the gut microbiome by preventing dysbiosis. In particular, it has been suggested that different lipids found in tomato exosomes lead to a decrease in *Clostridioides difficile*, which causes diarrhea in the intestine, and *Fusobacterium nucleatum* pathogens associated with periodontal diseases and inflammatory bowel diseases, as well as an increase in Gram-positive probiotic Lactobacillus species and, therefore, may be used in intestinal infections [78].

A study performed by Mu et al. focused on isolation and characterization of exosome-like nanoparticles from four edible plants (e.g., ginger, carrots, grapefruit, and grape). Further experiments showed that the isolated exosomes from four different sources were taken up by intestinal macrophages and stem cells and exerted beneficial effects on the recipient cells. Exosomes induced activation of Nrf2, HO-1, IL-10, and Wnt/transcription factor 4 (TCF4), which play crucial roles in antioxidation and anti-inflammation. Additionally, ginger-derived exosomes may restore intestinal homeostasis in the mice model. Taken all together, it has been postulated that PDEVs could expedite the gastrointestinal epithelial cell proliferation in inflammatory conditions [3].

##### Anti-Inflammatory Activity in the Skin

You and colleagues reported that exosomes isolated from cabbage (Cabex) and red cabbage (Rabex) efficiently provoked cell proliferation of HaCaT keratinocyte and RAW 264.7 cells and inhibited inflammation and apoptosis, thus protecting cells from stress [79]. EVs isolated from lemon juice were found to inhibit UV- and LPS-induced oxidative stress in dermal fibroblasts via the AhR/Nrf2 antioxidative pathway, accelerate wound closure, and exhibit antioxidant and anti-inflammatory effects in an in vivo zebrafish model [80]. In a study determining the effects of vesicles obtained from the bark of the *Aloe vera* plant on inflammation, oxidative stress, and burn wounds, RAW 264.7, THP-1 M0 macrophages, and HaCaT cells, which are cells associated with burn wounds, were used. It was reported that vesicles suppressed IL-1β, IL-6, and TNFα in RAW 264.7 and THP-1 M0 macrophages, and suppressed IL-1β and TNFα in HaCaT keratinocytes. It was also found that vesicles decreased myofibroblast differentiation and collagen contractile capacity, which were known to play a significant role in the process of burn wound closure [81].

##### Anti-Inflammatory Activity in Immune Cells

The anti-inflammatory effects of EVs derived from different plant species have been tested using numerous models with activated immune cells. In one of these studies, it has been reported that EVs originating from lemon can be used safely in primary T lymphocytes without changing the phenotype, and show anti-inflammatory effects in activated macrophages by inhibition of NF-kB and extracellular signal-regulated kinase (ERK) [82]. Immunosuppressive effects of celery-root-derived exosomes on phorbol 12-myristate, 13-acetate, and ionomycin-activated T lymphocytes and peripheral blood mononucleated cells have been reported [83]. Exosomes derived from Pueraria lobata, a medicinal herb and edible plant cultivated in China, have been shown to exhibit anti-inflammatory activity by changing the macrophage phenotype from M1 to M2 [84]. Anti-inflammatory effects of exosome-like nanoparticles isolated from papaya were tested in macrophage cells and a zebrafish model, and a decrease in polarization of neutrophils and macrophages, decrease in inflammatory cytokines, such as IL-1B and IL-6, and an increase in anti-inflammatory cytokines, such as IL-10, were determined [85]. It has been reported that EVs isolated from the fruits of *Solanum nigrum* L. suppress the inflammatory cytokine IL-6 in RAW 264.7 macrophage cells and that neral, a monoterpene compound, may play a role in these effects [86]. In another study, in vitro antioxidant and anti-inflammatory effects on the monocytic (THP-1) cell line have been reported for exosomes isolated from pomegranate [87].

##### Anti-Inflammatory Activity in Neurodegenerative Disorders and Ischemic Brain Diseases

Panax notoginseng, a medicinal plant with known beneficial effects on inflammation-related ischemic brain damage, was analyzed for the effectiveness of its EVs. The results revealed that, especially the lipids in panax-derived vesicles have been reported to reduce inflammation in microglia, protect the structure of the BBB, improve ischemic damage, and activate the survival pathway PI3K/Akt [88]. In another study, it was reported that the EVs derived from rhizoma root are rich in enzymes, such as NAD(P)H-quinone oxidoreductase, which may be a useful and potential drug for oxidative-stress-related neurodegenerative diseases, such as Alzheimer’s and Huntington’s disease [89].

##### Anti-Inflammatory Activity in Liver

In a study using garlic-derived EVs in a mouse model of acute liver failure induced by LPS/D-GalN combination therapy, the vesicles exhibited favorable effects on liver destruction. Decreased alanine aminotransferase (ALT) and aspartate aminotransferase (AST) liver enzymes, decreased inflammation markers, such as IL-1β, IL-6, and TNF-α, as well as decreased migration of monocyte macrophages to the liver by suppressing CC chemokine receptor type 2 (CCR2)/CC chemokine receptor type 5 (CCR5) were reported as positive changes observed in vesicle-treated mice. In addition, decreased hepatocyte apoptosis with garlic-derived EV treatment was confirmed by decreases in caspase 3, caspase 9, and bax, and an increase in bcl-2. In addition, vesicle administration reprogrammed metabolism in the liver and reversed the suppression of autophagy, which is involved in the development of liver injury [90].

##### Immune Stimulant Activity

Activation of immune cells by PDEVs has also been reported in the literature. It has been found that *Catharanthus roseus*-derived exosomes increase the immune activity and polarization of macrophages via TNF-α/NF-κB/PU.1 activation; thus, they can be used as immune stimulants after chemotherapy [91].

#### 3.5.2. Anticancer Properties

**Figure 4:**
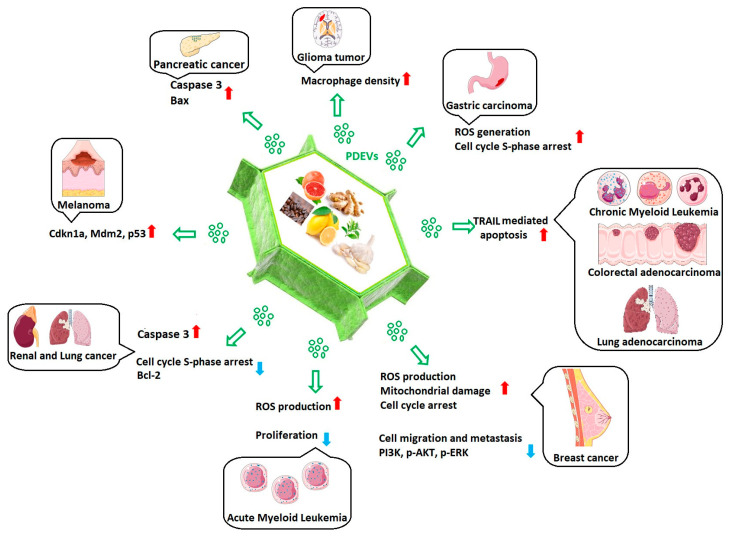
The promising anticancer effects of plant-derived extracellular vesicles (PDEVs) n various cancer models. PDEVs exhibit cytotoxic and anticancer activity against multiple tumor cell lines by inducing apoptosis via TRAIL activation, reactive oxygen species (ROS) production, caspase induction, mitochondrial damagee, cell cycle arrest, suppression of cell migration and metastasis.

Since their discovery, PDEVs have been tested for their biological effects in different cancer models, with very promising results (Figure 4). The potential role of PDEVs in alleviating various diseases, especially cancer, is regarded as an intriguing area for future research [92]. The PDEVs’ entrance into the cancer cells leads to modification of gene expression to lessen the phenotypes associated with cancer [58]. In line with this, Raimondo et al. indicated that exosomes derived from lemon juice (*Citrus limon* L.) prohibited cell proliferation in various tumor cell lines (e.g., human chronic myeloid leukemia cell line (LAMA84), colorectal adenocarcinoma cell line (SW480), and lung carcinoma cell line (A549)), thus exerting anticancer activity through activating a TRAIL (tumor necrosis factor-alpha-related apoptosis-inducing ligand)-mediated apoptotic cell death [93]. Recently, Yang and colleagues revealed that lemon-derived EVs (LDEVs) exert potential anticancer effects on gastric cancer cells through the generation of reactive oxygen species (ROS). Additionally, they reported that the apoptotic effect of LDEVs was mediated via cell cycle S-phase arrest. Further, LDEVs were regarded as safe EVs when applied to inhibit tumor growth in SGC-7901 tumor-bearing mice and could be retained in the GIT [32]. Another study was performed to investigate the anticancer effects of EVs derived from various plant saps from Korea, including *Dendropanax Morbifera* (DM), *Pinus densiflora* (PD), *Thuja occidentalis* (TO), and *Chamaecyparis obtusa* (CO), on normal, low metastatic, and malignant cells. The results of the study revealed that both DM-EVs and PDEVs exerted selective cytotoxic effects toward malignant breast and skin t-umor cells, with no cytotoxic effect on normal cells. Besides, the combination of DM-EVs and PDEVs resulted in improved cytotoxic activity against malignant breast and skin tumor cells. However, TO-EVs and CO-EVs were not cytotoxic upon most of the tumor cells [31]. In another study, Chen and colleagues assessed anticancer activity of EVs derived from fresh tea flowers (*Camellia sinensis* (L.) O. Kuntze). The isolated EVs were composed of polyphenols, flavonoids, functional proteins, and lipids. The tea flowers’ EVs were cytotoxic against breast cancer cells through ROS production. Elevated levels of ROS, in turn, led to the initiation of mitochondrial damage and cell cycle arrest. Furthermore, in vivo experiments illustrated that intravenous or oral administration of tea-flower-derived EVs accumulated in breast tumors and lung metastatic sites, though they prohibited breast cancer development and metastasis and modulated gut microbiota [44]. Recently, the anticancer activity of garlic-derived EVs (GEVs) was investigated on two cancer cell lines, A498 (renal cancerous cells) and A549 (lung cancer), as well as one normal cell line (HDF). The GEV treatment led to markedly reduced cancer cell viability, however, it did not cause a significant cytotoxic effect on the normal cell line. The GEVs exerted anticancer activity through the S-phase cell cycle arrest. Additionally, downregulation of anti-apoptotic Bcl-2 expression levels and increased caspase 3 activity are considered as other reasons for GEVs’ anticancer properties [94].

PDEVs have also been tested on various liver cancer models. EVs originating from the cannabis chemotype with high cannabidiol content have been reported to induce cell cycle arrest and the mitochondrial apoptosis pathway in HepG2 and Huh-7 hepatocellular carcinoma cell lines, while showing no toxic effect on human umbilical vein endothelial cells (HUVECs) [57]. It has been suggested that coffee-derived exosomes may protect against liver fibrosis and liver cancer by targeting Zinc Finger Protein 773 (ZNF773) and lysine N-methyltransferase 2C (KMT2C) genes through the miRNAs they carry [95]. Exosome-like nanostructures of tea leaf origin have been shown to suppress breast cancer and lead to apoptosis by remodeling the microbiota when administered orally to mice. Interestingly, while the same particles caused side effects, such as liver and kidney toxicity and inflammation, when administered intravenously, these side effects were not observed when administered orally and, therefore, they were recommended as oral nano-therapeutics [96]. In another study on breast cancer, a combination of local therapy and systemic doxorubicin therapy was tested in TNBC cells by loading exosomes originating from citrus lemon into hydrogels. The study also mimicked breast cancer in vivo with the use of 3D bioprinters, and the results showed that the combined therapy induced oxidative stress and suppressed cancer cell migration and metastasis. The same therapy showed growth-promoting and wound-closure-stimulating effects on fibroblasts to accelerate post-surgical healing [97]. The anticancer effects of EVs isolated from *Citrus lemon* L. on TNBC cells 4T1 and HCC-1806 were mediated by decreasing the phosphorylation of PI3K, AKT, and ERK proteins associated with survival [98]. In another recent study, the contents of ginger-, lemon-, grapefruit-, and grape-derived EVs were compared, and it was found that ginger-derived vesicles carried more gingerol and shogaol in the lipid profile compared to other vesicles and, therefore, the effects of ginger vesicles on melanoma cells were tested. It was reported that these vesicles exhibited significant anti-tumor activity in B16F10 melanoma cells and B16F10 tumor-bearing C57BL/6 mice, which involved the induction of Cdkn1a and Mdm2 genes and the p53 pathway in their apoptotic effects [43]. In another study on melanoma, EVs isolated from different Aloe species have been proposed to be used as photosensitizing, photodynamic agents in melanoma treatment due to their active anthraquinone compounds [59].

Piperlongumine-derived exosomes have been reported to induce cell death in retinoblastoma cells more effectively than known chemotherapy drugs, increasing caspase 3/7 activity and mitochondrial potential loss [68,99]. EVs isolated from basil have been reported to increase apoptotic bax and caspase 3 gene expression and decrease clonogenicity in pancreatic cancer cells [100]. Ginseng-derived nanoparticles have been reported to show effective targeting in C6 glioma models of BAlb/c and Wistar mice thanks to their capability to pass the BBB and increase M1 macrophage density in the microenvironment of the glioma tumor [101]. EVs were also tested in leukemia, a blood malignancy. EVs obtained from grapefruit, which contains high amounts of the powerful antioxidant ascorbic acid, were found to contain measurable levels of ascorbic acid, catalase, and glutathione. Vesicles were shown to exhibit a significant anti-proliferative effect in blast cells isolated from AML patients, as well as HL60, Hel, MV4-11, U937, and K562 cell lines. It has also been reported that vesicles stimulate cell death by increasing ROS in cancer cells, but not in normal hematopoietic pluripotent cells [53].

#### 3.5.3. Various Health-Promoting Effects of PDEVs

**Figure 5:**
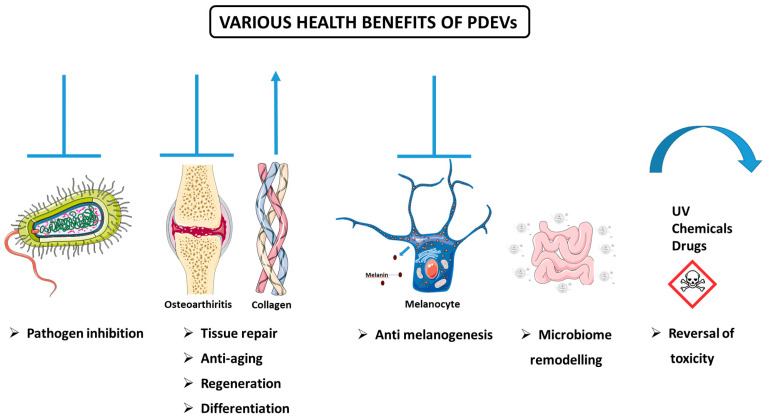
Effects of PDEVs on various pathological states. Protection against pathogens, anti-arthrithic and anti-melanogenesis activities, remodelling of the microbiome and reversal of chemical, drug and UV induced toxicity are among the versatile health effects of PDEVs.

In addition to the anticancer and anti-inflammatory properties of PDEVs, recent research has highlighted their potential to offer various health benefits (Figure 5). This section will discuss some of the studies that have been implemented thus far.

##### Defense Against Pathogenic Species

PDEVs exert an important role in the defense mechanisms of plants against pathogens. For instance, PDEVs derived from sunflower seedlings protect the plant from infections via defense proteins, which in turn modulate immune responses [31]. It has been evident that plants may inhibit pathogens and their virulence by EV-mediated shuttling of miRNAs. In line with this, Cai et al. exhibited that EVs produced by Arabidopsis cells transferred small RNAs into the fungal pathogen *Botrytis cinerea*. These small RNAs, which accumulated at the infection site, led to silencing of fungal virulence genes, thus prohibiting pathogenicity [102].

PDEVs’ ability to combat pathogens is probably fulfilled through different mechanisms, including inhibition of pathogen adhesion and augmenting immune responses. In an in vivo study conducted by Sundaram and colleagues, the anti-infection features of ginger exosomal lipids and miRNAs were assessed in a mice model. The isolated exosomes prohibited *Porphyromonas gingivalis* (a Gram-negative bacteria involved in chronic periodontitis) pathogenicity through reduction of FimA (fimbriae A) expression, which in turn led to inhibition of *P. gingivalis* attachment to oral epithelial cells. Besides, reduced recruitment of macrophages, leukocytes, and cluster of differentiation (CD) 3 cells into the oral tissue microenvironment was observed. Furthermore, their results showed that drinking water supplemented with ginger exosomes resulted in increased bone mineral density in naive mice [103].

##### Anti-Arthritic, Regenerative, Proliferative, and Differentiation Activities

An in vivo experiment’s findings revealed that EVs formulated from pepper (*Bhut jolokia*)—capsicum indigenous to Northeast India—exerts good topical anti-arthritic activity by reducing arthritis-associated inflammations and nociception in the rat model [104].

Another study investigated the potential of PDEVs in the treatment of osteoarthritis by stimulating the repair of cartilage tissue. In the study, *S. lycopersicum-* and *C. limon*-derived vesicles were isolated, and their effects on mesenchymal stem cells to chondrocyte differentiation were investigated. The results showed that chondrocyte-specific cartilage regeneration markers aggrecan (ACAN), SRY-Box Transcription Factor 9 (SOX9), and cartilage oligomeric matrix protein (COMP) were increased in tomato-derived vesicle-treated cells, while COL2 and COLXI proteins were increased in cartilage extracellular matrix. The opposite effects were determined for lemon-derived vesicles [105]. Ginseng-derived exosome-like vesicles have been reported to inhibit bone resorption by preventing osteoclast differentiation [106]. Similar anti-osteoporosis effects have been reported for exosomes originating from another plant, the yam of the dioscorea family [107]. Ginger-derived EVs are involved in the healing of ringworm by induction of hair follicle proliferation and suppression of inflammation, with 13 specific compounds associated with hair proliferation [56].

Moreover, dermatologic studies indicated the advantages of PDEVs. Consistent with this, Sahin and colleagues, for the first time, studied the *Triticum aestivum* (known as wheat, one of the main cereal crops)-derived exosomes in wound closure via in vitro approaches. The results indicated that extracted exosomes exerted significant proliferative and migratory effects on different (endothelial, epithelial, and fibroblast) skin cells. Further, wheat exosome treatment significantly increased the expression levels of collagen type I mRNA [108].

Exosomes obtained from beet juice have been reported to exhibit regenerative effects by stimulating collagen synthesis in the skin [109]. EVs isolated from *Aloe saponaria* have been shown to inhibit the chronicity of skin wounds and increase the formation of HUVEC capillary tubes and exhibit positive effects on wound healing [110]. Nanovesicles from the Goji berry plant, whose effects on muscle and bone health have been previously reported, may reduce aging-related muscle loss through activation of the AMPK/sirtuin 1 (SIRT1)/peroxisome proliferator-activated receptor-γ coactivator 1-α (PGC1α) pathway and changes in sugar metabolism and oxidative phosphorylation in a mouse model [69]. It has been suggested that exosomes isolated from *Cissus quadrangularis* reduce oxidative stress in MC3T3-E1 cells, increase proliferation and wound closure in human mesenchymal stem cells, and stimulate the differentiation of human mesenchymal stem cells into osteoblasts and C2C12 myoblasts, and with these effects, they can be used as therapeutics and drug carriers in bone disease [111].

##### Anti-Melanogenesis Activity

Numerous studies have shown that EVs of plant origin may be effective against hyperpigmentation. The inhibitory effects of leaf-derived (LEVs) and stem-derived extracellular vesicles (SEVs) of *Dendropanax morbifera* on melanin production were examined by Lee and colleagues, and their data indicated that the aforementioned EVs reduced melanin content and tyrosinase (TYR) activity in the B16BL6 mouse melanoma cell line in a concentration-dependent manner. Furthermore, LEVs reversed the expression of melanogenesis-related genes and proteins (e.g., microphthalmia-associated transcription factor (MITF), TYR, and tyrosinase-related proteins (TRP-1 and TRP-2)). LEVs inhibited the formation of melanin better than arbutin (a TYR inhibitor that served as a positive control), and thus could be utilized in hyperpigmentation treatment [33]. Furthermore, it has been reported that EVs isolated from *Ecklonia cava* plant exhibited anti-melanogenesis effects in keratinocyte and animal skin models treated with UV light together with Phlorotannin, and that suppression of oxidative stress and inhibition of the nucleotide-binding domain, leucine-rich-containing family, pyrin domain-containing-3 (NLRP3), and IL18 inflammasome play a role in these effects [112]. Edelweiss, an endangered plant known for its positive effects on the skin, was exposed to LED light in callus culture, and it was determined that this exposure led to a significant increase in flavonoid and phenolic contents and secondary metabolites of EVs isolated from the plant. The vesicles suppressed melanin production by showing a whitening effect in α-MSH-stimulated B16F10 cells and increased filaggrin (FLG), aquaporin 3 (AQP3), and COL1 proteins, with skin structural functions in fibroblast cells [113]. In another study, the miRNA contents of exosome-like nanoparticles obtained from *Atractylodes lancea* rhizome were examined in α-melanocyte-stimulating hormone (α-MSH)-treated B16-F10 melanoma cells. It was reported that the nanoparticles decreased the gene levels of microphthalmia-associated transcription factor (Mitf), tyrosinase, tyrosinase-related protein 1, and DOPA chromium tautomerase, which are involved in melanogenesis, and thus have therapeutic potential in diseases characterized by excessive melanin production [114].

##### Anti-Aging Activity

PDEVs have also emerged as promising tools for their anti-aging effects. Vesicles isolated from the *Ecklonia cava* plant have been found to reduce oxidative stress and aging by increasing HSP70 expression and decreasing inflammatory TNF-α, mitogen-activated protein kinase (MAPK), and NF-κB expression in keratinocyte cells in vitro and in aging mouse models in vivo [115]. Exosomes originating from the medicinal mushroom *Phellinus linteus* have also been reported to have beneficial effects against UV-induced aging [116].

##### Remodeling of the Microbiome

PDEVs also exert their biological effects by remodeling the microbiome. In one study, researchers investigated the impact of dietary plant-based miRNAs on the gut microbiome population in a mice model deficient in miR-146a. As the evidence suggests, loss of miR-146a in animals results in impaired gut health and an altered microbiome composition. Their results indicated that a plant diet rich in miR-146a can fine-tune microbiome composition in miR-146a-deficient mice compared to the control group [117]. In another study conducted by Teng and colleagues, the crosstalk between dietary EVs miRNAs with gut microbiota was investigated. They demonstrated that ginger EVs were selectively taken up by gut bacteria, resulting in the interaction of EVs’ RNAs with a panel of low-grade glioma (LGG) genes, which led to the alternation of the composition of the gut microbiota by affecting the growth of other gut bacteria [41].

It has been reported that exosomes isolated from cranberries exhibit positive effects in premature ovarian failure and reduce ovarian granulosa cell death, and intestinal microbiome changes play a role in these effects [118]. Another study reported that EVs originating from watermelon affect intestinal secretion into the placenta and suggested that they may be used to improve fetal growth and placental function [119]. It has also been suggested that PDEVs may exert anti-depression activity through their effects on the microbiome. It has been reported that administration of *Lepidium meyenii*-derived EVs to stressed mice reduces the abundant Enterococcus, Lactobacillus, and Escherichia Shigella bacteria in their feces and leads to changes in the metabolism of amino acids and biotin used in serotonin production. Through these changes, vesicles have been shown to suppress depression by providing neuronal plasticity through the GTP cell division control protein 42 homolog (Cdc42)/ERK and tropomyosin receptor kinase B (TrkB)/p-AKT pathways [120].

##### Reversal of Toxicity

PDEVs have also been reported to exhibit protective effects against toxicity induced by drugs, chemicals, and alcohol. Nanovesicles isolated from the roots of the *Pueraria lobata* plant carrying the puerarin isoflavone were examined for alcohol intoxication effects. In female and male mice administered certain doses of ethanol, it reduced the liver and blood levels of alcohol by affecting the enzymes metabolizing alcohol and inhibiting alcohol-induced ferroptosis [121]. In another study, the effects of beta vulgaris-derived exosomes on the heart were investigated in doxorubicin-treated C57BL/6N mice. Doxorubicin, a potent chemotherapy agent, unfortunately causes cardiac toxicity. Exosomes isolated from beta vulgaris were reported to reverse the toxic effects of doxorubicin on the heart without damaging organs, such as the liver, spleen, and kidney. These effects were largely mediated by antioxidant mechanisms and suppression of ferroptosis [122]. In another study, exosomes isolated from ginseng have been reported to play a role in the reversal of cisplatin-induced cardiotoxicity. It was determined that oxidative stress and inflammation were suppressed by MAPK inhibition in exosome-treated cardiomyocytes compared to untreated cells and markers, such as lactate dehydrogenase, cardiac troponin, and creatine kinase, which increase during cardiac damage, were also reduced [123]. Furthermore, *Momordica charantia*-derived nanovesicles have been reported to exert protective effects on radiation-induced cardiomyocyte injury, mediated by stimulation of survival and prevention of apoptosis, protection of mitochondria, and reduction of oxidative stress [124]. In a study on exosome-like nanoparticles originating from green onions, these particles were found to protect hippocampal cells against glutamate-induced toxicity. Oxidative stress, lipid peroxidation, and intracellular Fe levels induced by glutamate exposure in hippocampal cells were reversed by the application of nanoparticles. Modulation of proteins involved in iron metabolism and increased glutathione peroxidase 4 (GPX4) played a role in these effects [125]. Vesicles from the medicinal plants *S. sclarea* and *S. dominica* were tested in an in vitro model of Parkinson’s disease, and a reduction in autooxidation and oxidative stress was seen in SH-SY5Y cells treated with 6-hydroxydopamine, a neurotoxin [126].

##### Other Health Effects

Apple-derived EVs have been reported to suppress the sodium-dependent bile acid uptake transporter (ABST) and exhibit positive effects on chronic constipation and cholestasis [127]. There are also various studies on the positive effects of plant-derived nanovesicles on the liver. In one of these studies, the effects of nanovesicles isolated from tangerine peel on hepatic steatosis associated with type 2 diabetes were investigated in a mutant diabetic mouse model, and the effects of nanovesicles on peroxisome proliferator-activated receptor alpha (PPAR-α), PGC1α, mitochondrial uncoupling protein 1 (UCP1), and PR domain-containing 16 (PRDM16) were analyzed. It has been reported that they increase the expression of genes associated with fatty acid oxidation, such as carnitine palmitoyltransferase 1 (CPT1) and carnitine palmitoyltransferase 2 (CPT2), decrease the expression of genes involved in lipogenesis, such as sterol regulatory element binding protein 1c (SREBP1-c), CD36, acetyl-CoA carboxylase (ACC), liver X receptor (LXR-α), PPAR-γ, and CCAAT enhancer binding protein (CEBPα), and thus reduce fat droplet accumulation in the liver. It has also been reported that vesicles lead to remodeling in intestinal microbiota, decreasing harmful bacteria, such as Lachnospiraceae and Desulfovibrionaceae, and increasing the density of beneficial bacteria, such as Lactobacillaceae and Muribaculaceae [128].

#### 3.5.4. PDEVs and Bio-Macromolecule/Drug Delivery

**Figure 6:**
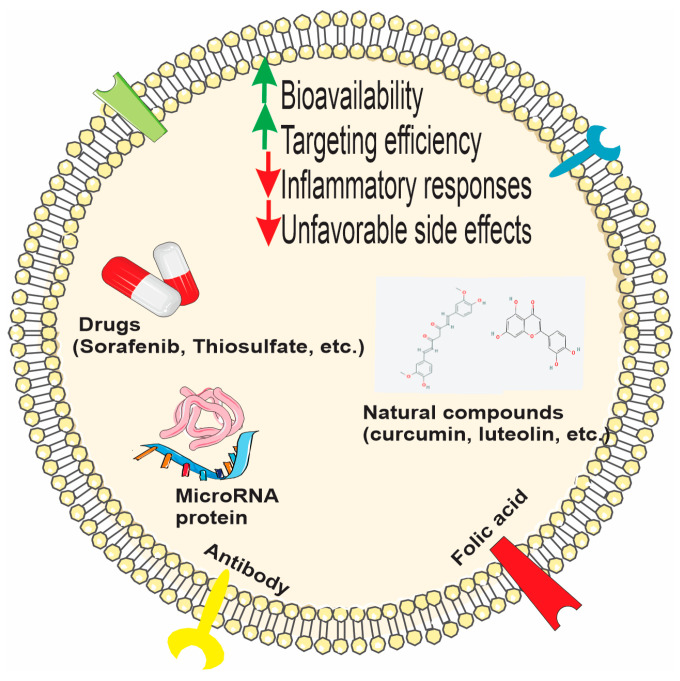
The role of PDEVs as versatile, natural carriers for delivering a wide range of therapeutic agents (methotrexate and thiosulfate), biomolecules, such as microRNAs, proteins, and peptides, as well as natural compounds, like curcumin, tangeretin, and luteolin. Surface modification techniques, such as coating PDEVs with antibodies or folic acid, enable targeted delivery to specific cells or tissues, improving therapeutic efficacy. PDEVs offer enhanced drug solubility, bioavailability, and targeted delivery, potentially reducing toxicity and dosage requirements.

Nowadays, the formation of nontoxic natural nanovectors from PDEVs or PDEV-derived lipids is considered as an alternative approach in nanomedicine and drug delivery [7,129,130]. Multiple factors, like poor bioavailability, limited water solubility, and probable side effects or interactions with drugs, may affect the health-promoting effects of bio-active compounds, which are called extra-nutritional constituents (such as polyphenols, vitamins, or polyunsaturated fatty acids (PUFAs)) [15]. Maintenance of the high efficiency of transfection of molecules/drugs while delivering to the target cells, despite mammalian barriers, requires vehicles to overcome unfavorable off-target effects or lack of a host immune response [131]. Moreover, low cytotoxicity, low production cost, and high production yield make PDEVs promising candidates in this field [79]. The specific advantage of PDEVs is their capability to bind hydrophobic agents, which in turn may increase their bioavailability and their subsequent cellular uptake [3,61,132]. Moreover, PDEVs potentially can pass through the BBB; nevertheless, they cannot cross the placental barrier [61,133]. For all these reasons, PDEVs, both in their natural and possibly modified forms, have attracted attention as possible drug carriers and vaccine platforms (Figure 6).

##### PDEVs as Carriers of Natural Compounds and Drugs

Several studies exploited grapefruit-derived EVs (GDEVs) for drug delivery. In this regard, Wang et al. incorporated methotrexate (MTX)—an immunosuppressant and anti-inflammatory drug—into GDEVs. They transferred MTX-GDEVs into mice model, resulting in a significant reduction in MTX toxicity in comparison to the free MTX, as well as augmentation of MTX’s therapeutic properties in DSS-induced mouse colitis. They suggested that GDEVs might serve as a suitable candidate for oral delivery of small molecules or drugs, with the aim of attenuating inflammatory responses in human disease [42]. In another study, it was shown that in addition to their high phenolic and flavonoid content, vesicles isolated from *Citrus reticulata* Blanco cv have the potential to effectively encapsulate and transport tangeretin, a flavone. The vesicles showed significant antioxidant and anti-inflammatory activity in an LPS-induced macrophage inflammation model [55]. Exosomes isolated from tomato fruit were loaded with curcumin, and significant anti-inflammatory activity was obtained [134]. Again, in another study using curcumin, nanovesicles with very high curcumin-carrying capacity were designed from turmeric by quantitative nanoflow cytometry. In vitro anti-lipogenesis, lipolysis, and apoptosis induction in 3T3-L1 cells were observed, and more effective anti-obesity activity was obtained in vivo compared to free curcumin treatment [135]. In another study, luteolin, a flavonoid with solubility, stability, and bioavailability problems, was loaded into exosome-like nanovesicles obtained from sesame leaves, and more effective antioxidant and anti-inflammatory activity in macrophages was obtained compared to exosomes and free luteolin [136].

Due to the potential side effects and bioavailability problems of sodium thiosulfate, which is an approved drug for vascular calcification, its application as a different drug formulation was considered, and its effects were investigated by loading into grapefruit-derived vesicles. The EVs used for this purpose were modified with hydroxyapatite crystal binding peptide, and their effects as a nanodrug were investigated. In mice exposed to calcifying medium, it was reported that the applied nanodrug accumulated in calcification sites and suppressed calcification in vascular smooth muscle cells, triggered the anti-inflammatory M2 macrophage profilerepressed the transition from a contractile to osteogenic profile in the vascular phenotype, increased bone quality, and did not cause any toxicity [137]. PDEVs have also been tested as carriers of sorafenib, a drug that for years has suffered from low oral bioavailability, side effects, and off-target effects. It was reported that sorafenib-loaded kiwifruit-derived vesicles did not cause hemolysis in mice, accumulated mainly in the liver 24 h after administration, and were efficiently taken up intracellularly by HepG2 cells, but their uptake was lower in fetal hepatocytes LO2, thus reducing off-target and side effects. It was also reported that sorafenib-loaded vesicles showed significant cytotoxic activity in HepG2 cells and reduced tumor weight and volume in an orthotopic HepG2 liver cancer xenograft nude mouse model [138].

##### Bioengineered PDEVs and Hybrid Systems for Targeted Therapies

Grapefruit-derived lipids were used to design nanovectors for chemotherapeutic agents’ delivery. For improving in vivo targeting efficiency, they co-delivered therapeutic agents with folic acid into folate receptor-expressing cells. They observed that grapefruit-derived nanovectors have the potential to hinder tumor growth in CT26- and SW620-cell-derived tumors in mice models. In addition to the aforementioned results, they reported that the toxicity of these nanovectors was decreased when compared to the nanoparticles formulated by synthetic lipids. Also, they confirmed that nanovectors did not pass the placental barrier after intravenous injection into pregnant mice, proposing that they may serve as an advantageous tool for drug delivery during pregnancy [135]. Later, the same group indicated that GDEVs coated with inflammatory-related receptor-enriched membranes of activated leukocytes (IGDEVs) efficiently targeted inflammatory tumor tissues in different models of inflammatory disease in mice. Besides, the IGDEVs effectively prohibited the tumor growth in vivo through improving the chemotherapeutic activity and the inflammatory effects of DSS-induced mouse colitis [139]. In another study, EVs isolated from grapefruit were either fused with gum-derived mesenchymal stem cells and/or loaded with rRNA with a specific transcription inhibitor, CX5461. Researchers reported that high-affinity binding of CCL20 to CC chemokine receptor type 6 (CCR6) on T helper 17 (Th17) cells is involved in the migration of T cells to the site of inflammation. Gingival cells overexpressing CCR6 and mesenchymal stem cells originating from these cells were bio-designed by lentiviral intervention. Hybrid exosomes alone, without drug loading, suppressed ROS and activated CD3+ and CD4+ T cell proliferation in LPS-induced RAW 264.7 cells. Furthermore, hybrid exosomes loaded with CX5461 have also been reported to stimulate the polarization of macrophages from M1 to M2 phenotype in vitro, reduce ROS levels in macrophages in HaCaT cells, suppress T cell proliferation and the janus kinase signal transduction and activation of transcription (JAK-STAT) pathway, and effectively target lesioned tissues and exhibit anti-inflammatory effects in psoriasis and atopic dermatitis mouse models, which are among the common autoimmune skin diseases [140]. Another nanovector designed by Zhang et al. was from ginger-derived lipids for doxorubicin delivery in order to treat colon cancer. The ginger-derived EVs were taken up by colon cancer cells and significantly reduced cancer cell proliferation and induced apoptosis [141]. In another recent study, curcuma-derived extracellular vesicles (CNVs) were loaded with doxorubicin and coated on their surface with an antibody capable of binding to death receptor 5, specific for senescent cancer cells. The study demonstrated that the vesicles stimulated apoptosis and suppressed angiogenesis in aging cancer cells both in vitro and in vivo [142].

##### PDEVs as Carriers of Small RNA

EVs of plant origin have also been investigated for their activity as small RNA carriers. It was suggested that EVs originating from broccoli, apple, orange, and pomegranate protect the miRNAs they carry from RNAase degradation and can be used as carriers of different miRNAs. In the study, it was reported that vesicles, especially those originating from broccoli, showed cytotoxic activity on cancer cells both alone and when loaded with specific miRNAs [143]. In another study performed by Zhuang et al., they utilized GDEVs for miR17 delivery to the brain tumor of the mice model as a noninvasive therapeutic approach. The results of their research demonstrated that GDEVs coated with folic acid (FA- GDEVs) efficiently targeted the folate-receptor-positive GL-26 brain tumor. Furthermore, reduced toxicity of polyethyleneimine and enhanced transmittance of RNA were observed in FA-GDEV-coated polyethyleneimine (FA-pGDEVs). Intranasal administration of FA-pGDEVs/miR17 in mice led to delayed brain tumor growth [144]. *Panax ginseng*-derived exosomes were coated with neutrophil membranes isolated from mice, and bioengineered vesicles were used as miRNA carriers and tested in a sepsis model of acute lung injury. As a result of miRNA chip analysis between healthy and sepsis patients, miR182-5p, which exhibits low expression in sepsis patients, was loaded into exosomes. It was reported that exosomes reduced lipid peroxidation, slowed lung damage, and suppressed inflammatory cytokines by inducing antioxidant enzymes in mice, in which sepsis induced acute lung injury andreppressed NADPH oxidase 4 (NOX4), which is the target of miRNA 182-5p and involved in mitochondrial functions in LPS-stimulated MLE-12 lung epithelial cells [145]. In another study investigating the use of EVs in RNA interference studies, tangerine juice-derived vesicles were loaded with phospholipase DDHD1-siRNA, a target in colon cancer, using the electroporation method. It was reported that DDHD1 siRNA-loaded vesicles provided 60% suppression of the target gene in SW480 COLXI on cancer cells [146]. In another study, bioengineered ginger-derived vesicles were loaded with TNF-α siRNA, and positive results were obtained by repairing the intestinal barrier and remodeling the intestinal microbiota in an ulcerative colitis model [147]. PDEVs were also tested for their efficiencies in transferring antagomiRs, a class of chemically engineered oligonucleotides to silence endogenous microRNAs. In a study using green-tea-derived exosomes as antagomiR carriers, significant results were obtained for the prevention of aortic dissection, thereby developing a form resistant to stable transport and enzyme elimination of antagomiR, leading to vascular remodeling through matrix metalloproteinase 9 (MMP9) and myocyte enhancer factor 2D (Mef2D) pathways [148].

##### PDEVs as Carriers of Peptides and Proteins

Tomato- and grapefruit-derived vesicles were tested for their transporter efficiency, and it was found that yield of grapefruit-derived vesicles transferred HSP70 protein into glioma cells with higher efficiency compared to tomato-derived vesicles [149]. EVs of plant origin have also been investigated as possible carrier systems for cosmetic applications. It has been reported that the use of PDEVs for the application of peptides, which are inherently unstable and exhibit limited absorption from the skin, exhibits advantages, such as much more effective passage through the skin barrier compared to free peptides [150].

##### PDEVs as Vaccine Platforms

EVs of plant origin are also of interest in improving the stability, transfer, transport, and shelf-life properties, which are important factors in the development of vaccines. It has been reported that EVs obtained from *Citrus sinensis* oranges have the potential to be loaded with mRNA and used as a vaccine encoding the S2 protein subunit of SARS-CoV-2, which can be administered through the mouth or nose. Furthermore, the mRNA carried by these vesicles is protected against enzymatic degradation and induces a significant immune response. It was also found that lyophilized mRNA-carrying vesicles can remain stable at room temperature for one year. These results showed that EVs of orange origin may provide significant advantages in vaccine development [151,152].

#### 3.5.5. PDEVs in Clinic

In light of the encouraging outcomes observed in the studies employing PDEVs, a series of clinical trials have been initiated to further investigate their potential benefits. To date, a series of clinical trials have been conducted; however, the comprehensive results have yet to be disclosed. In this regard, a clinical trial (NCT01294072) was started in 2011 at the University of Louisville to assess the capacity of plant-derived exosomes to facilitate curcumin delivery into normal and cancerous colon tissue. This study is currently in the recruiting phase. In 2012, researchers at the University of Louisville initiated another clinical trial (NCT01668849) to investigate the anti-inflammatory activity of grape-derived exosomes in decreasing the prevalence of oral mucositis in patients with head and neck tumors during radiation and chemotherapy. However, the results of this study have not been published. Another clinical trial (NCT04879810) was initiated in 2018 at the University of Louisville. The objective of this study was to assess the anti-inflammatory effects of ginger- or curcumin-derived exosomes on the symptoms and disease score in patients with refractory IBD. The study was completed, however, the results are not yet available. In 2019, a clinical trial (NCT03493984) was conducted to study the effects of the ginger- or Aloe-derived exosomes on polycystic ovary syndrome (PCOS); nevertheless, the study was subsequently withdrawn. A clinical trial (IRCT202001270462822N53) was initiated in 2024 at the Tehran University of Medical Sciences (Iran) to evaluate the efficacy of plant-derived, Wharton jelly-derived, and minoxidil treatments for hair loss in affected individuals.

Hence, promising results from several studies have gained growing attention from scientists in the field of applying PDEVs in treating various diseases. Further understanding of PDEVs’ diverse beneficial effects on improving health is required.

## 4. Final Remarks and Conclusions

Plant-derived extracellular vesicles (PDEVs) are natural carriers of biomolecules (proteins, lipids, nucleic acids, and metabolites) into mammalian cells, potentially benefiting disease treatment and healthcare. The effects of PDEVs on pathological conditions, such as oxidative stress, inflammation, and cancer, have been investigated in various in vitro and in vivo studies, which have generally reported favorable effects of PDEVs. PDEVs have significant potential in overcoming drug bioavailability barriers. Their large surface area, strong targeting ability, and slow degradation properties reduce off-target effects, improve therapeutic selectivity, and make them effective at tissue compartments. Existing literature findings revealed that PDEVs show no toxicity or immunogenic effects in vivo, a major advantage for their potential use in drug delivery and therapy. They are biocompatible and environmentally friendly, with no harmful pathogens for humans, and can be extracted from abundant plants. Additionally, they have the potential for incorporating antimicrobial, anti-inflammatory, and anticancer properties through their miRNA and secondary metabolite contents. In addition, PDEVs can be engineered to deliver therapeutic compounds to specific sites by increasing the solubility and bioavailability. The PDEVs’ content release has a great role in intercellular and cross-kingdom communications through gene regulation in the recipient cells.

Despite all these positive features, PDEVs have various limitations. The lack of standardized extraction methods and exosomal markers for the isolation and identification of PDEVs lead to variations in the size and content of vesicles, and hence difficulties in their characterization. Another limitation is that the bioactive compounds in PDEVs may vary depending on the plant species, plant part, and isolation technique, which hinders the identification of reliable therapeutic mechanisms. The biological state of the recipient cell may also be a factor for different responses to PDEV treatments and may complicate standard therapy. Existing studies do not provide clear information on the distribution, metabolism, and cellular uptake of PDEVs in the human body, and further clarifying studies are needed in this area. Although studies have shown that vesicles can transport various components, such as miRNAs, to the target site with their stability intact, further studies are still needed to clarify their stability and metabolic routes. In addition, the mechanisms mediating the specific targeting of certain vesicles to specific tissue sites need to be further elucidated.

We can conclude that the limitations of PDEVs stem from the uncertainties regarding their structure and biology, such as the identity and stability of their specific biocomponents, their metabolic transformation following ingestion, and the biological mechanisms they use for specific tissue targeting. Clarification of these issues in further research studies is vital to maximize the therapeutic efficiency and health-promoting potential of PDEVs.

## Data Availability

Not applicable.

## Design and methodology

The literature search for this review was conducted using the keywords “plant-derived extracellular vesicle”, “exosome biogenesis”, “plant vesicle lipid content”, “plant vesicle protein content”, “plant vesicle metabolite”and the primary database, PubMed. The majority of the studies included in the review were published between 2020 and 2025, ensuring that the findings reflect the most recent developments in the field. The remaining literature consists of seminal works, which laid the groundwork for research on plant-derived extracellular vesicles. The review is mainly focused on research studies, but reviews that address important points in this field are also used. Only peer-reviewed scholarly publications were included, and non-peer-reviewed sources were excluded from the analysis.

## Abbreviations

AMPK	Adenosine monophosphate-activated protein kinase
BBB	Blood–brain barrier
CAC	Colitis-associated cancer
DC	Dendritic cell
DSS	Dextran sodium sulfate
DOX	Doxorubicin
EV	Extracellular vesicle
FimA	Fimbriae A
GIT	Gastrointestinal tract
HO-1	Heme oxygenase-1
HSP	Heat shock protein
IAV	Influenza A virus
IBS	Irritable bowel syndrome
IEC	Intestinal epithelial cell
IL	Interleukin
LDLRAP1	Low-density lipoprotein receptor adaptor protein 1
LPS	Lipopolysaccharide
miRNA	microRNA
mRNA	messenger RNA
mTOR	Mammalian target of rapamycin
MV	Membrane vesicles
MVB	Multivesicular body
NF-κB	Nuclear factor-κB
Nrf-2	Nuclear factor erythroid 2-related factor 2
NS1	Nonstructural protein 1
PA	Phosphatidic acid
PB2	Polymerase basic protein 2
PCOS	Polycystic ovary syndrome
PDEV	Plant-derived extracellular vesicle
PI3K	Phosphoinositide 3-kinases
ROS	Reactive oxygen species
TLR	Toll-like receptor
TNBC	Triple-negative breast cancer
TNF-α	Tumor necrosis factor alpha
TRAIL	TNF-related apoptosis-inducing ligand
TYR	Tyrosine
GC	Guanine-cytosine
ITGB3	Integrin beta-3
PDGFRB	Platelet-derived growth factor receptor beta
Lgr5	Leucine-rich repeat-containing G protein-coupled receptor 5
TCF4	Transcription factor 4
ERK	Extracellular signal-regulated kinase
PI3K	Phosphatidylinositol 3 kinase
AKT	Protein kinase B
ALT	Alanine aminotransferase
AST	Aspartate aminotransferase
CCR2	CC chemokine receptor type 2
CCR5	CC chemokine receptor type 5
CCR6	CC chemokine receptor type 6
ZNF773	Zinc finger protein 773
KMT2C	Lysine N-methyltransferase 2C
ACAN	Aggrecan
SOX9	SRY-Box Transcription Factor 9
COMP	Cartilage oligomeric matrix protein
COL	Collagen
HUVEC	Human umbilical vein endothelial cells
SIRT1	Sirtuin 1
PGC1α	Peroxisome proliferator-activated receptor-γ coactivator 1-α
TRP	Tyrosinase-related protein-1
NLRP3	Nucleotide-binding domain, leucine-rich-containing family, pyrin domain-containing-3
FLG	Filaggrin
AQP3	Aquaporin 3
MAPK	Mitogen-activated protein kinase
LGG	Low-grade glioma
Cdc42	Cell division control protein 42 homolog
Trkb	Tropomyosin receptor kinase B
GPX4	Glutathione peroxidase 4
ASBT	Apical sodium-dependent bile acid transporter
PPAR-α	Peroxisome proliferator-activated receptor alpha
UCP1	Mitochondrial uncoupling protein 1
PRDM16	PR domain-containing 16
CPT	Carnitine palmitoyltransferase
SREBP1-c	Sterol regulatory element binding protein 1c
CD	Cluster of differentiation
ACC	Acetyl-CoA carboxylase
LXR	Liver X receptor
Cebpα	CCAAT enhancer binding protein
Th17	T helper 17
JAK-STAT	Janus kinase signal transduction and activation of transcription
NOX4	NADPH oxidase 4
MLE-12	Mouse lung type II epithelial cell line
MMP	Matrix metalloproteinase
Mef2D	Myocyte enhancer factor 2D
